# Pagetoid dyskeratosis in dermatopathology^☆^^☆☆^

**DOI:** 10.1016/j.abd.2020.12.006

**Published:** 2021-06-01

**Authors:** Tauana Ogata Coelho da Rocha, Fernanda Gonçalves Moya, Vívian Moreira Vilella, Rute Facchini Lellis

**Affiliations:** a Department of Dermatology, Complexo Hospitalar Heliópolis, São Paulo, SP, Brazil; b Department of Pathology, Hospital das Clínicas, Faculdade de Medicina, Universidade de São Paulo, São Paulo, SP, Brazil; c Private Clinic, Araçatuba, SP, Brazil; d Department of Pathology, Irmandade da Santa Casa de Misericórdia de São Paulo, São Paulo, SP, Brazil; e Department of Pathology, AC Camargo Câncer Center, São Paulo, SP, Brazil

**Keywords:** Clear epithelial cells, Dyskeratosis, Pagetoid dyskeratosis

## Abstract

Currently, pagetoid dyskeratosis is believed to involve an accelerated keratinization process, possibly induced by mechanical trauma. It represents, in almost its totality, incidental histological findings of specific cells, except when it occurs in the hands, where it usually occurs simultaneously with skin lesions and local dyschromia. These are large, rounded keratinocytes, with pale cytoplasm and a pyknotic nucleus surrounded by a clear halo, which can be easily mistaken by other skin diseases. Its etiology is not completely elucidated, and the correct identification of this entity can be of great importance in the differential diagnosis of skin disorders and the understanding of the keratinization process of the epidermis.

## Introduction

Pagetoid dyskeratosis (PD) is an incidental histopathological finding that can be observed in skin biopsies of different lesions and different locations.^1^ Despite representing just a finding, PD in the hands, unlike other sites, can occur simultaneously with dermatological manifestations, especially papules with local dyschromia.^1^

PD cells were and still are described as larger than normal keratinocytes, with a rounded, pale cytoplasm and a pyknotic nucleus surrounded by a clear halo, similar to Paget's disease cells, which is the cause for the name of this condition.^2^, ^3^

Although easily identified, they can be misdiagnosed, due to the similarity of these cells with those in other diseases such as Paget cells, Toker cells of the nipple and koilocytes.^2^, ^4^ Therefore, the identification of this condition can be of great importance in the differential diagnosis with other skin diseases, in addition to helping understand the keratinization process of the epidermis, as subsequently demonstrated.^4^

## Case report

**Figure 1:**
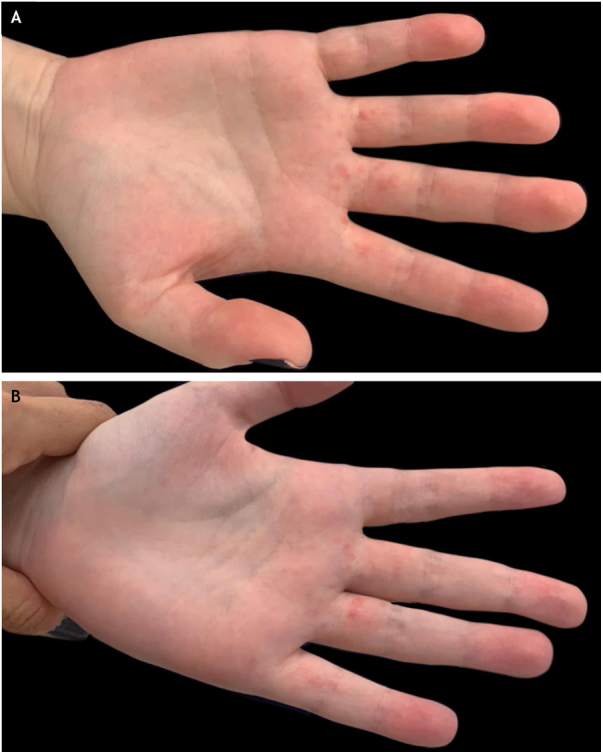
Clinical presentation of pagetoid dyskeratosis on the hands. (A) Small erythematous papules on the palmar surface of proximal phalanges of 2^nd^, 3^rd^ and 4^th^ fingers of the right hand and (B) 3^rd^, 4^th^ and 5^th^ fingers of the left hand.

A 19-year-old female patient reported that she had small erythematous papules on the palmar surface of the proximal phalanges of the 2^nd^, 3^rd^ and 4^th^ fingers of the right hand and 3^rd^, 4^th^ and 5^th^ fingers of the left hand for a year (Fig. 1). She reported intermittent lesions, with spontaneous improvement one week after their onset, with new worsening after constant use of pens, palm-sweating, when snapping the fingers with the palms touching each other or when the hands were clenched for long periods. She reported mild local pain and denied any other associated symptoms.

**Figure 2:**
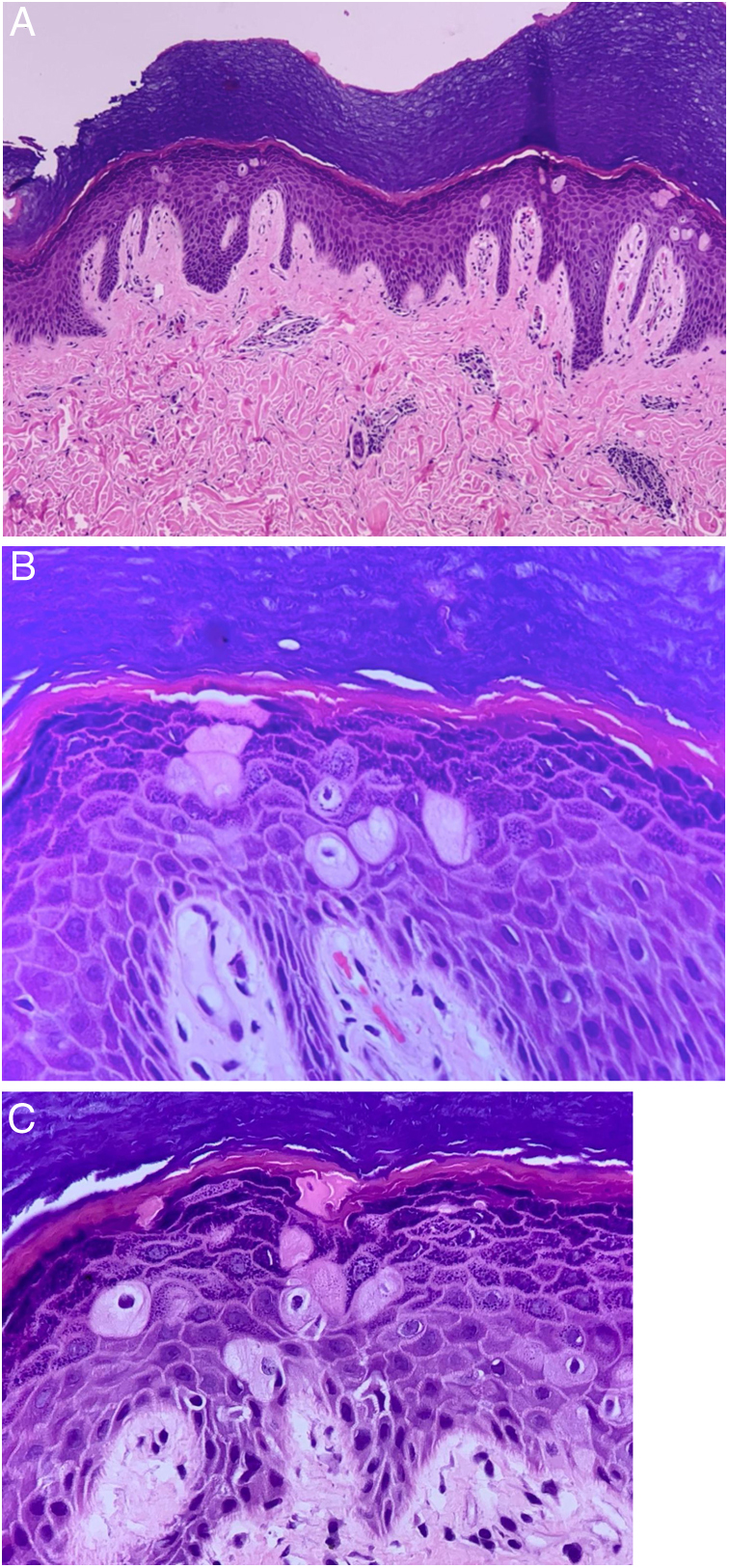
Pagetoid dyskeratosis. (A) Hyperkeratosis, hypergranulosis and mild acanthosis. Bulky cells with large eosinophilic cytoplasm, pyknotic nucleus, and clear perinuclear halo amid keratinocytes. Congested superficial and deep dermis and discrete perivascular lymphohistiocytic infiltrate (Hematoxylin & eosin, ×100). (B) and (C) Dyskeratotic cells are well seen (Hematoxylin & eosin, ×400).

Due to the lack of lesion improvement, a skin biopsy was performed, which showed hyperkeratosis, hypergranulosis and mild acanthosis; amidst the keratinocytes, bulky cells with a large and eosinophilic cytoplasm, pyknotic nucleus, and perinuclear clear halo were observed; the superficial and deep dermis were congested and showed discrete perivascular lymphohistiocytic infiltrate (Fig. 2). The anatomoclinical correlation led to the conclusion that the patient had pagetoid dyskeratosis of the hands.

## Discussion

In 1980, Mehregan assessed 15 patients with varied skin lesions, all of which contained clear epithelial cells characterized by a pyknotic nucleus surrounded by a clear halo and a pale cytoplasm, without keratohyalin granules and intercellular connections between them and neighboring keratinocytes.^3^ These cells were found in the spinous layer above the basal layer and were well recognized in the granular layer, becoming unrecognizable in the stratum corneum, after undergoing dissolution of the nucleus during the keratinization process.^3^ At the end of their report, they concluded that the presence of these cells in specimens from unrelated conditions suggested an artifact, possibly related to moisture or other unknown physical factors.^3^

Although PD was originally considered an artifact, it was Tschen et al, in 1988, who first suggested that PD could represent a reactive process of early keratinization.^2^ For them, the definition of the epidermis as comprising two cell compartments – one being the basal layer, which represented undifferentiated proliferative cells and the other being the suprabasal cells undergoing differentiation – was an exaggerated simplification, as it is possible to find cells undergoing mitosis in the suprabasal layer, as well as cells in the basal layer expressing suprabasal keratins.^2^ Therefore, instead of considering clear epithelial cells as an artifact that repeated itself throughout the specimens, Tschen et al. speculated that these cells were probably a small part of the normal keratinocyte population in the basal layer that underwent mitosis and developed their own cytoplasmic characteristics, possibly resulting from their proliferation after stimuli such as trauma, and thus, could be induced by friction.^2^, ^5^ Since then, PD has been recognized as a dyskeratotic process and classified among the epidermal maturation defects (it seems to be an accelerated keratinization process).^6^

In 2015, Santos-Briz et al. conducted a study to assess the occurence of pagetoid dyskeratotic cells, considered to this day an incidental finding in skin biopsies, without clinical significance.^4^ They analyzed all biopsies performed over one year at the Department of Dermatology at Hospital Universitario de Salamanca – Spain, totaling 3,565 biopsies, of which 80 had PD cells (2.24%).^2^, ^4^, ^7^ Although the patients did not report significant mechanical trauma, most cases of PD appeared in exophytic lesions.^4^ Moreover, in 80% of polypoid lesions, PD cells were located in the middle and upper layers of the epidermis, which would be more commonly exposed to physical trauma, such as friction.^4^ Hyperkeratosis (89.3%) and hypergranulosis (82.5%) were found in most biopsies, which also indirectly indicates friction.^4^ In addition, Pique-Duran et al.^8^ demonstrated that the axillary location is frequent for PD, which also corroborates the theory that moisture and friction are related to its presence, findings that are consistent with previous data.^8^

These cells have been reported as an incidental histological finding in several cutaneous diseases that are unrelated to each other, such as melanocytic nevi, lichen simplex chronicus, acrochordons, seborrheic keratoses, among others, and in several different anatomical locations.^4^, ^5^, ^9^ However, contrary to other sites where PD is a finding, pagetoid dyskeratosis on the hands usually presents with discrete changes at the site, as in the case of the abovementioned patient, in addition to being quite uncommon, with only 10 cases reported to date.^9^, ^10^

In summary, PD, with the exception of the hands, is an incidental, but not uncommon, finding present in more than 2% of skin biopsies, which can be interpreted as a type of accelerated keratinization in a keratinocyte population induced to proliferate after traumatic stimuli. Pathologists should be aware of this finding to avoid misdiagnosing it as other conditions, such as mammary and extramammary Paget's disease, squamous cell carcinoma *in situ*, epidermotropic metastases, superficial spreading pagetoid malignant melanoma, clear cell papulosis, and koilocytosis, which represent the differential diagnoses of the disease. A better understanding of PD could probably improve the present understanding of the keratinization process and other epidermal keratinization disorders.^2^, ^4^

## Financial support

None declared.

## Authors’ contributions

Tauana Ogata Coelho da Rocha: Drafting and editing of the manuscript; collection, analysis, and interpretation of data; critical review of the literature.

Fernanda Gonçalves Moya: Effective participation in research orientation; intellectual participation in propaedeutic and/or therapeutic conduct of the studied cases.

Vívian Moreira Vilella: Statistical analysis; collection, analysis, and interpretation of data.

Rute Facchini Lellis: Approval of the final version of the manuscript; design and planning of the study; critical review of the manuscript.

## Conflicts of interest

None declared.
